# Leaf extracts from *Dendropanax morbifera* Léveille mitigate mercury-induced reduction of spatial memory, as well as cell proliferation, and neuroblast differentiation in rat dentate gyrus

**DOI:** 10.1186/s12906-019-2508-6

**Published:** 2019-05-02

**Authors:** Woosuk Kim, Dae Young Yoo, Hyo Young Jung, Jong Whi Kim, Kyu Ri Hahn, Hyun Jung Kwon, Miyoung Yoo, Sanghee Lee, Sung Min Nam, Yeo Sung Yoon, Dae Won Kim, In Koo Hwang

**Affiliations:** 1 Department of Anatomy and Cell Biology, College of Veterinary Medicine, and Research Institute for Veterinary Science, Seoul National University, Seoul, 08826 South Korea; 2 Department of Anatomy, College of Medicine, Soonchunhyang University, Cheonan, Chungcheongnam 31151 South Korea; 3 Department of Biochemistry and Molecular Biology, Research Institute of Oral Sciences, College of Dentistry, Gangneung-Wonju National University, Gangneung, 25457 South Korea; 4 Korea Food Research Institute, Jeollabuk-do, 55365 South Korea; 5 Department of Anatomy, College of Veterinary Medicine, Konkuk University, Seoul, 05030 South Korea

**Keywords:** *Dendropanax morbifera* extract, Mercury, Morris water maze, Neurogenesis, Hippocampus

## Abstract

**Background:**

The brain is susceptible to methylmercury toxicity, which causes irreversible damage to neurons and glia and the leaf extract *Dendropanax morbifera* Léveille (DML) has various biological functions in the nervous system. In this study, we examined the effects of DML on mercury-induced proliferating cells and differentiated neuroblasts.

**Methods:**

Dimethylmercury (5 μg/kg) and galantamine (5 mg/kg) was administered intraperitoneally and/or DML (100 mg/kg) was orally to 7-week-old rats every day for 36 days. One hour after the treatment, novel object recognition test was examined. In addition, spatial probe tests were conducted on the 6th day after 5 days of continuous training in the Morris swim maze. Thereafter, the rats were euthanized for immunohistochemical staining analysis with Ki67 and doublecortin and measurement for acetylcholinesterase (AChE) activity.

**Results:**

Dimethylmercury-treated rats showed reduced discrimination index in novel object recognition test and took longer to find the platform than did control group. Compared with dimethylmercury treatment alone, supplementation with DML or galatamine significantly ameliorated the reduction of discrimination index and reduced the time spent to find the platform. In addition, the number of platform crossings was lower in the dimethylmercury-treated group than in controls, while the administration of DML or galantamine significantly increased the number of crossings than did dimethylmercury treatment alone. Proliferating cells and differentiated neuroblasts, assessed by Ki67 and doublecortin immunohistochemical staining was significantly decreased in the dimethylmercury treated group versus controls. Supplementation with DML or galantamine significantly increased the number of proliferating cells and differentiated neuroblasts in the dentate gyrus. In addition, treatment with dimethylmercury significantly increased AChE activity in hippocampal homogenates, while treatment with dimethylmercury+DML or dimethylmercury+galantamine significantly ameliorated this increase.

**Conclusions:**

These results suggest that DML may be a functional food that improves dimethylmercury-induced memory impairment and ameliorates dimethylmercury-induced reduction in proliferating cells and differentiated neuroblasts, and demonstrates corresponding activation of AChE activity in the dentate gyrus.

## Background

Heavy metals such as mercury, lead, and cadmium are hazardous because they are bioaccumulated and biomagnified as they ascend the food chain. In particular, dimethylmercury (MeHg), the most common cause of intoxication in humans [1], accumulates with the consumption of fish, including long-lived predatory species such as sharks and tuna. Absorbed MeHg enters the bloodstream, easily crosses the blood-brain barrier [2, 3], and is distributed to the brain, including the hippocampus and cerebellum [4]. Accumulated evidences demonstrated that Sprague-Dawley rats were most widely used animal models for MeHg toxicity in the brain. In addition, impairments of neurite outgrowth and cell migration was pronounced in the cells derived from male fetuses compared to cells from females [5]. Developmental MeHg exposure has been shown to impair memory during puberty [6, 7]; cognitive functions are impaired and ultrastructural abnormalities are observed in the dentate gyrus of adult rats exposed to MeHg [8]. In addition, treatment with MeHg impairs the cellular excitability and synaptic transmission by blocking the blocks calcium and sodium channels in rat hippocampal slices, while it does not affect the synaptic plasticity [9]. MeHg was found to reduce cell proliferation in the hippocampus at postnatal day 7 (P7), and significantly decrease granule cell layer population at P21 [6].

The hippocampus is a critical brain region involved in memory formation. Most of the granule cells in the hippocampal dentate gyrus are generated prenatally and intensive proliferation occurs during the first postnatal week, with differentiation into neurons occurring up to the third postnatal week [10, 11]. In the adult brain, the active cells located in the subgranular zone of the dentate gyrus are able to proliferate, migrate to granule cell layer, and differentiate into neuroblasts. Neuroblasts are integrated into granule cells and contribute to learning and memory processes [12]. Hence, the facilitation of cell proliferation and neuroblast differentiation can be targets to promote hippocampal function and regenerative processes in neurological disorders such as Alzheimer’s disease.

Medicinal plants have been widely investigated because of their ability to cure various disease states and maintain health with little to no adverse effects [13, 14]. *Dendropanax morbifera* is an endemic and evergreen plant in the Southern Korea and its leaves, stems, and roots are widely used in folk medicine for the treatment of skin problems and headache [15]. *Dendropanax morbifera* was also shown to ameliorate neuronal damage in an animal model of Parkinson’s disease by reducing neuroinflammation [16] and memory deficits in a chemically-induced aging model by decreasing pro-inflammatory cytokine levels in the hippocampus [17]. In addition, *Dendropanax morbifera* also alleviated hippocampal impairment in cadmium- and mercury-induced neurotoxicity rats [18, 19] and reversed the calcium-induced reduction of proliferating cells and differentiated neuroblasts in the hippocampus [20]. Previously, we demonstrated that MeHg increased oxidative stress in the hippocampus, with the administration of an extract of *Dendropanax morbifera* leaves (DML) significantly ameliorating this increase [19]. Treatment with DML shows fewer side effects such as cutaneous atrophy and erythema compared to that in the repeated dexamethasone use [21]. In addition, randomized, double-blinded trial conducted by our colleagues demonstrates there are minimal adverse effects in humans [22]. However, no studies have been reported on the preventive potentials of *Dendropanax morbifera* on MeHg-induced memory deficits, decreases in proliferating cells and differentiated neuroblasts in the hippocampus. The present study, therefore, investigated the effects of DML and MeHg on hippocampal memory, proliferating cells and differentiated neuroblasts in the dentate gyrus of the hippocampus.

## Methods

### Experimental animals

We purchased the male Sprague-Dawley rats (6 weeks of age) from Orient Bio Inc. (Seongnam, South Korea). Two animals were housed per cage at constant temperature (22 ± 2 °C) and humidity (60 ± 5%) on a 12/12 h light/dark cycle with ad libitum access to food and water. The experimental protocols and ethics were approved the Institutional Animal Care and Use Committee of Seoul National University (SNU-130911-4 and SNU-151104-1). All experiments were conducted with an effort to minimize the number of animals used and the physiological stress caused by the procedures employed. All experimental procedures were conducted according to ARRIVE guidelines [23].

### Preparation of DML

DML were obtained by ethanol extraction as previously described [19]. Briefly, DML was extracted with 80% ethanol, and then refluxed three times for 2 h. Centrifugation was conducted at 10,000×*g* for 30 min to remove the insoluble materials and a powder was obtained after concentration and freeze-drying.

### Quantification of phenolic compounds in DML by HPLC

HPLC analysis of DML was performed described by Hyun et al. [24]. DML sample was dissolved in 80% methanol solution and filtered with 0.2-μm syringe filter (TITAN, nylon) for high-performance liquid chromatography (HPLC).

The Thermo ACELLA HPLC (Thermoscientific, Waltham, MA, USA) system was used in this study with C18, 2.1 × 100 mm, 2.6-μm column (Thermo Accucore). Flow rate of solvent was set with 500 μL/min and every 4 μL of the sample was injected with 25 min of runtime. Rutin, chlorogenic acid, (+)-catechin, ferulic acid, quercetin, and myricetin were dissolved in methanol and the concentration of these compounds was measured by calculation of peak areas of samples with the calibration curve of the standards.

### Administration of MeHg and DML

Animals were randomly divided into four groups (*n* = 15/group): 1) distilled water- and saline-treated control group (control group), 2) distilled water- and MeHg-treated group, 3) MeHg and DML (MeHg+DML)-treated group, and 4) MeHg and galantamine (MeHg+GAL)-treated group. Distilled water, 100 mg/kg DML, and 5 mg/kg GAL was orally administered to 7-week-old rats once daily for 36 days, while saline and 5 μg/kg MeHg was treated intraperitoneally. GAL inhibits acetylcholinesterase activity and shows ameliorative effects on the reduction of hippocampal neurogenesis after cadmium exposure [20] and scopolamine toxicity [25, 26].

### Novel object recognition test

The test was performed as described in our previous study with minor modifications [20]. Briefly, the testing apparatus was made with black acryl with 80 × 60 × 40 cm open box. The objects were purchased with metallic to avoid the displacements by the rats due to their weight.

On 34th day of treatment with DML, GAL, or MeHg, 1 h after treatment, rats from each group (*n* = 5 per group) were freed to explore the apparatus for 2 min for adaption. The next day, 2-min training and testing trials were conducted 1 h following treatment with DML, GAL, or MeHg. During the training trial, animals were allowed to explore two identical objects placed in opposite corners of the apparatus. After 1 h interval, one familiar object was replaced into new (novel) object and rats were exposed to the objects during testing trial. Exploration time was measured when the nose of rat is located in no more than 2 cm of objects. Total spent time was calculated in both trials to explore the familiar and new objects. To elucidate the rats’ discrimination of two objects, discrimination index was also calculated by ratio of exploration time in each object vs. total time spent exploring the two objects in testing trial.

### Morris water maze test

On 29th day of treatment with DML, GAL, or MeHg, the Morris water maze test was conducted with Charles V Voorhees & Michael T Williams method. Animals were placed in the water maze pool, which is divided into four quadrants (A, B, C, and D). Each animal (*n* = 10 per group) was randomly trained once in each quadrant, for a total of four times a day, for 5 consecutive days. Escape latency was automatically checked as the time elapsed to find the platform and escape from the water.

On 35th day of treatment with DML, GAL, or MeHg, 1 h after treatment, the platform was removed and the rats entered the water from a different quadrant. In this spatial probe test, which measures learning and memory ability, rats were observed and recorded for 30 s to measure the time spent and the number of crossings in the quadrant at the original platform position.

### Tissue processing

One day after Morris water maze test, 1 h after treatment with DML, GAL, or MeHg, rats (*n* = 10 in each group) were euthanized with overdose of 2 g/kg of urethane (Sigma-Aldrich, St. Louis, MO, USA). Transcardiac perfusion was performed with 0.1 M phosphate-buffered saline (PBS, pH 7.4), and followed by 4% paraformaldehyde in 0.1 M PBS (pH 7.4), as previously described [20, 27]. Serial coronal hippocampal sections (30 μm) were made using a cryostat (Leica, Wetzlar, Germany) located 3.00 and 4.08 mm posterior to bregma in the rat brain atlas [28].

### Immunohistochemistry

Tissue sections, located 90 μm apart, were used for immunohistochemical staining. Briefly, primary antibodies were used as follows; rabbit anti-Ki67 antibody (1:1000; Abcam, Cambridge, UK) and rabbit anti-doublecortin (DCX) antibody (1:5000; Abcam). The antigen-antibody-horseradish peroxidase complex is allowed to react with a 3,3′-diaminobenzidine tetrachloride (Sigma) substrate for staining.

### Data analysis

DCX immunoreactivity in hippocampal dentate gyrus was analyzed ImageJ software v. 1.50 (National Institutes of Health, Bethesda, MD, USA), as described previously [20]. Briefly, the intensity of DCX immunoreactivity was measured according to pixel resolution with 256 Gy levels and relative optical density (ROD) was calculated by the following formula: ROD = log_10_ (256/mean gray level).

The number of Ki67- and DCX-immunoreactive cells were counted in dentate gyrus using an analysis system (OPTIMAS software version 6.5; CyberMetrics® Corporation, Phoenix, AZ, USA; magnification, 100×) as described previously [20]. The image was converted to a gray-scale image and Ki67-positive nuclei and DCX-immunoreactive neuroblasts were automatically selected according to the intensity of the immunohistochemical staining.

### Measurement of AChE activity in the hippocampus

One day after novel object recognition test, 1 h after treatment with DML, GAL, or MeHg, rats (*n* = 5 in each group) were euthanized to measure AChE activity in synaptosomes as described previously [20]. Briefly, hippocampi in each animal were quickly removed from the brain and a quick-freeze was conducted in liquid nitrogen for 15 min. Synaptosomes in hippocampal tissues were freshly isolated with discontinuous Percoll gradient [29]. The AChE enzyme activity was measured with the formation of the yellow anion, 5,5′-dithio-bis-acid nitrobenzoic [30].

### Statistical analysis

The data were expressed as mean with standard error of the mean or standard deviation. Mean differences among the groups were analyzed with Student *t*-test or one-way analyses of variance followed by Bonferroni’s post-hoc test using GraphPad Prism 5.01 software (La Jolla, CA, USA). Statistical significance was set at *p* < 0.05.

## Results

### Quantification of phenolic compounds in DML

For standardization and to observe the functional components of DML, most abundant 6 phenolic compounds in DML described by Hyun et al. [24] were measured. Rutin was most abundantly observed in the DML, while myricetin was lowest content in the DML. Chlorogenic acid, (+)-catechin, and ferulic acid was observed with 7.2–18.3 μg/g in DML (Table 1).

**Table 1:** Contents of phenolic compounds in DML are analyzed by HPLC method. Among these 6 phenolic compounds, rutin is the highest content and myricetin is lowest in DML. Values are mean ± standard deviation (*n* = 3)

No.	Phenolic compound	Concentration (μg/g)
1	Rutin	26.8 ± 0.4
2	Chlorogenic acid	18.3 ± 0.6
3	(+)-Catechin	14.9 ± 0.1
4	Ferulic acid	12.8 ± 0.4
5	Quercetin	7.2 ± 0.3
6	Myricetin	4.9 ± 0.2

### Effects of MeHg and DML on Morris water maze test

**Fig. 1:**
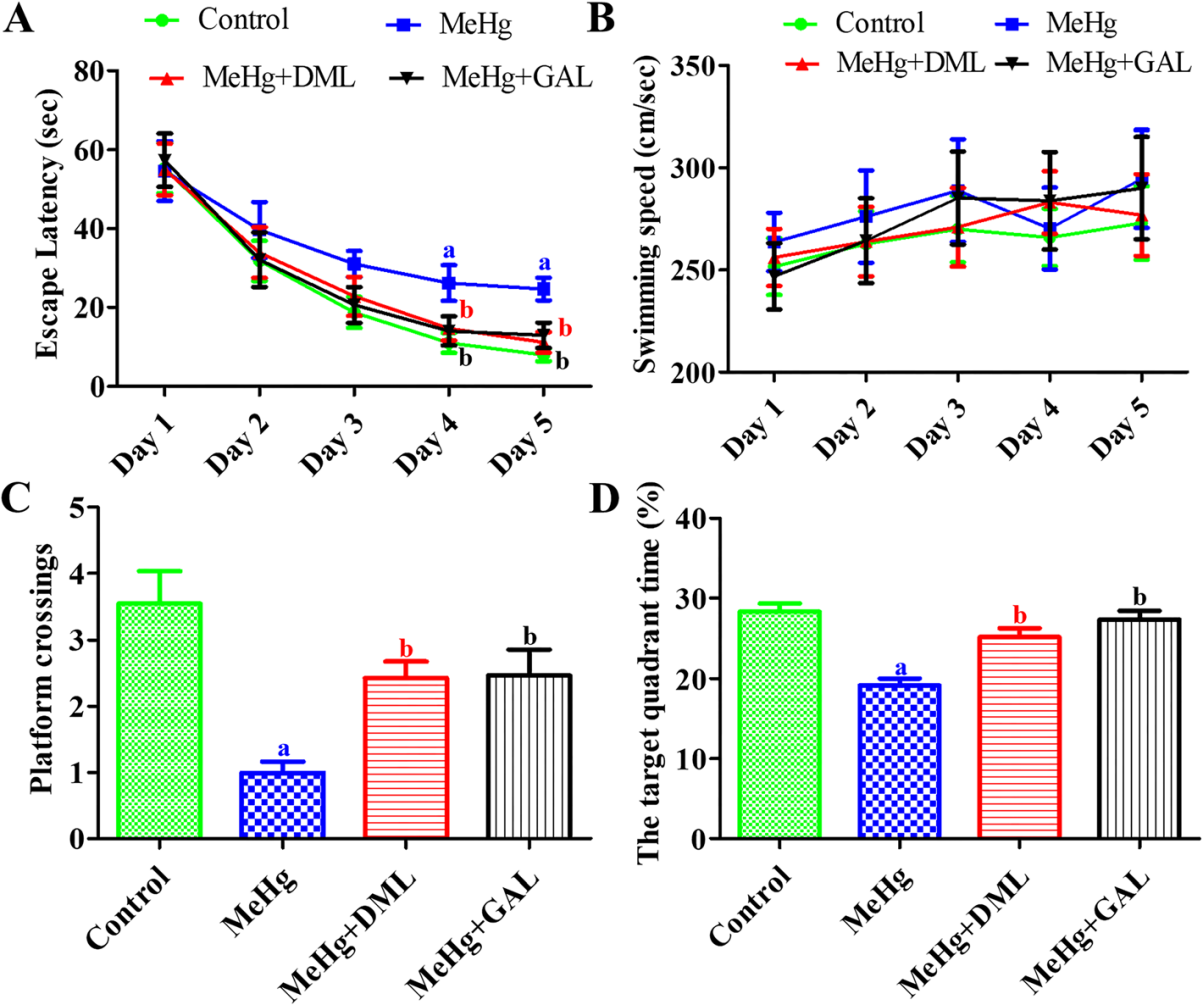
Escape latency training trials (**a**), average speed (cm/sec) (**b**), frequency of target crossing (**c**), and time spent in the correct quadrant (**d**) of the control group, dimethylmercury (MeHg)-treated group, *Dendropanax morbifera* leaf extract treated group with MeHg (MeHg + DML), and galantamine (GAL) treated group with MeHg (MeHg + GAL) in the Morris water maze task (*n* = 10; ^a^*p* < 0.05, vs. control group; ^b^*p* < MeHg-treated group). Values are mean ± standard error of the mean. Note that escape latency is highest in the MeHg group on days 4 and 5 and it is significantly decreased in the MeHg+DML and MeHg+GAL group on the days 4 and 5. In addition, on the day 6, number of crossings and target quadrant time is significantly decreased in the MeHg group compared to that in the control group and they significantly ameliorated in MeHg+DML and MeHg+GAL group

In all groups, escape latency progressively decreased from days 1 to 5, although the swimming speed did not show any significant differences among groups. There was a significantly higher escape latency in the MeHg-treated group compared the control group on days 4 and 5. MeHg+DML-treated rats showed a significant reduction in escape latency on the days 4 and 5 compared to the MeHg-treated group. In addition, MeHg+GAL-treated rats had similar escape latency in all experimental periods with that in the MeHg+DML-treated rats (Fig. 1a and b).

On day 6, the number of crossings at the original platform location and the target quadrant time were measured. MeHg-treated rats showed a significant reduction in the number of crossings and target quadrant time compared to the control group. However, MeHg+DML and MeHg+GAL treatment significantly reversed these effects (Fig. 1c and d).

### Effects of MeHg and DML on novel object recognition test

**Fig. 2:**
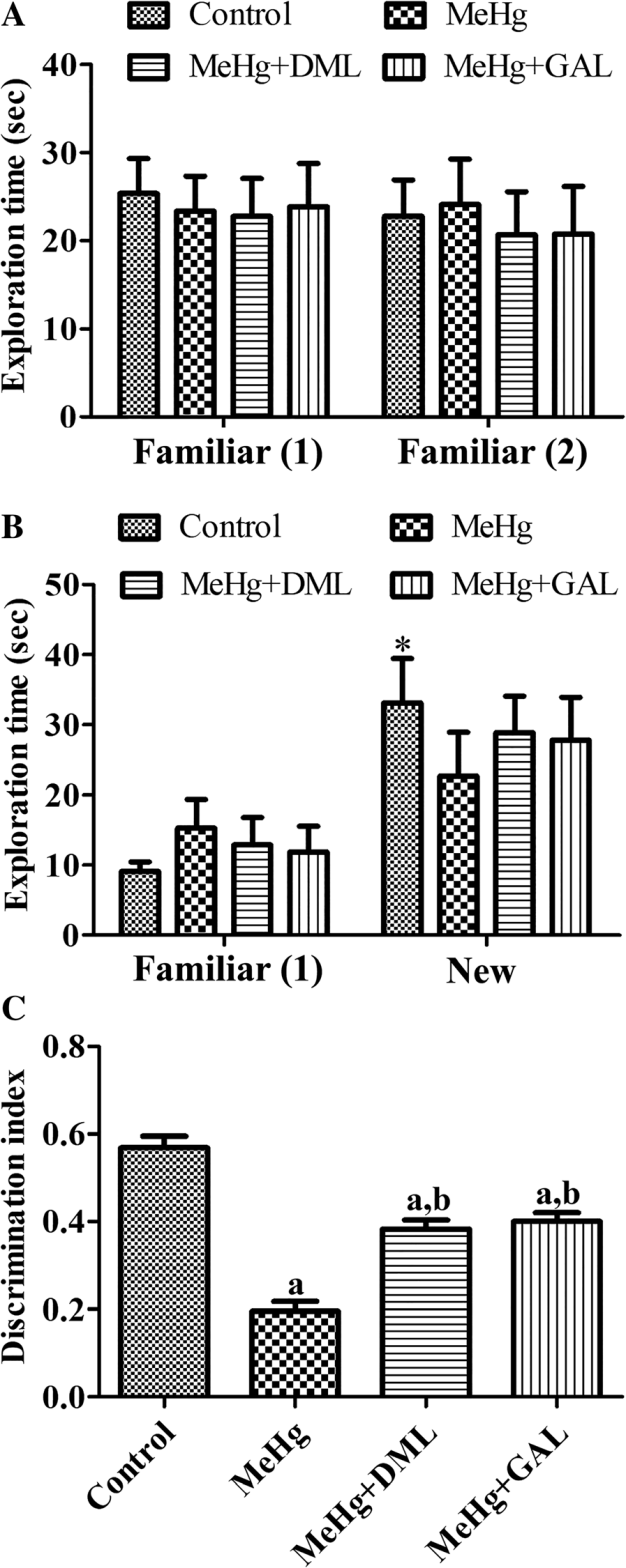
Exploration time in training (A) and testing trials (B), and discrimination index (C) in the control group, dimethylmercury (MeHg)-treated group, *Dendropanax morbifera* leaf extract treated group with MeHg (MeHg + DML), and galantamine (GAL) treated group with MeHg (MeHg + GAL) (*n* = 5 per group; ^*^p < 0.05, vs. familiar object; ^a^*p* < 0.05, vs. control group; ^b^*p* < MeHg-treated group). All data are shown as % exploration time ± standard error of the mean. Note that training trial does not show any remarkable differences on exploration time. In contrast, control group shows increased exploration time in new object compared to that in the MeHg group, while exploration time is decreased in MeHg group. In the MeHg+DML and MeHg+GAL groups, exploration time in new object is increased. In addition, discrimination index is significantly decreased in the MeHg group compared to that in the control group. In the MeHg+DML and MeHg+GAL group, discrimination index is remarkably increased compared to that in the MeHg group

In training trial, all animals did not show any significant differences on exploration time in two same familiar objects (Fig. 2a). In the testing trial, rats in control group showed significant increases in exploration time in new object than in familiar object. MeHg-treated rats showed a reduction in exploration time in new object compared to that in the control group, and there were no significant differences on exploration time spent between familiar and new object. Rats in MeHg+DML- and MeHg+GAL-treated groups spent much more time in new object than in the familiar object compared to that in the MeHg-treated group. However, statistical significance was not detected between groups (Fig. 2b). Discrimination index showed significant differences between groups. In the MeHg-treated group, discrimination index was significantly decreased compared to that in the control group. In the MeHg+DML-treated group, discrimination index was significantly increased compared to that in the MeHg group, although discrimination index in this group was significantly lower than in the control group. In the MeHg+GAL-treated group, discrimination index was similarly observed compared to that in the MeHg+DML-treated group (Fig. 2c).

### Effects of MeHg and DML on cell proliferation in the dentate gyrus

**Fig. 3:**
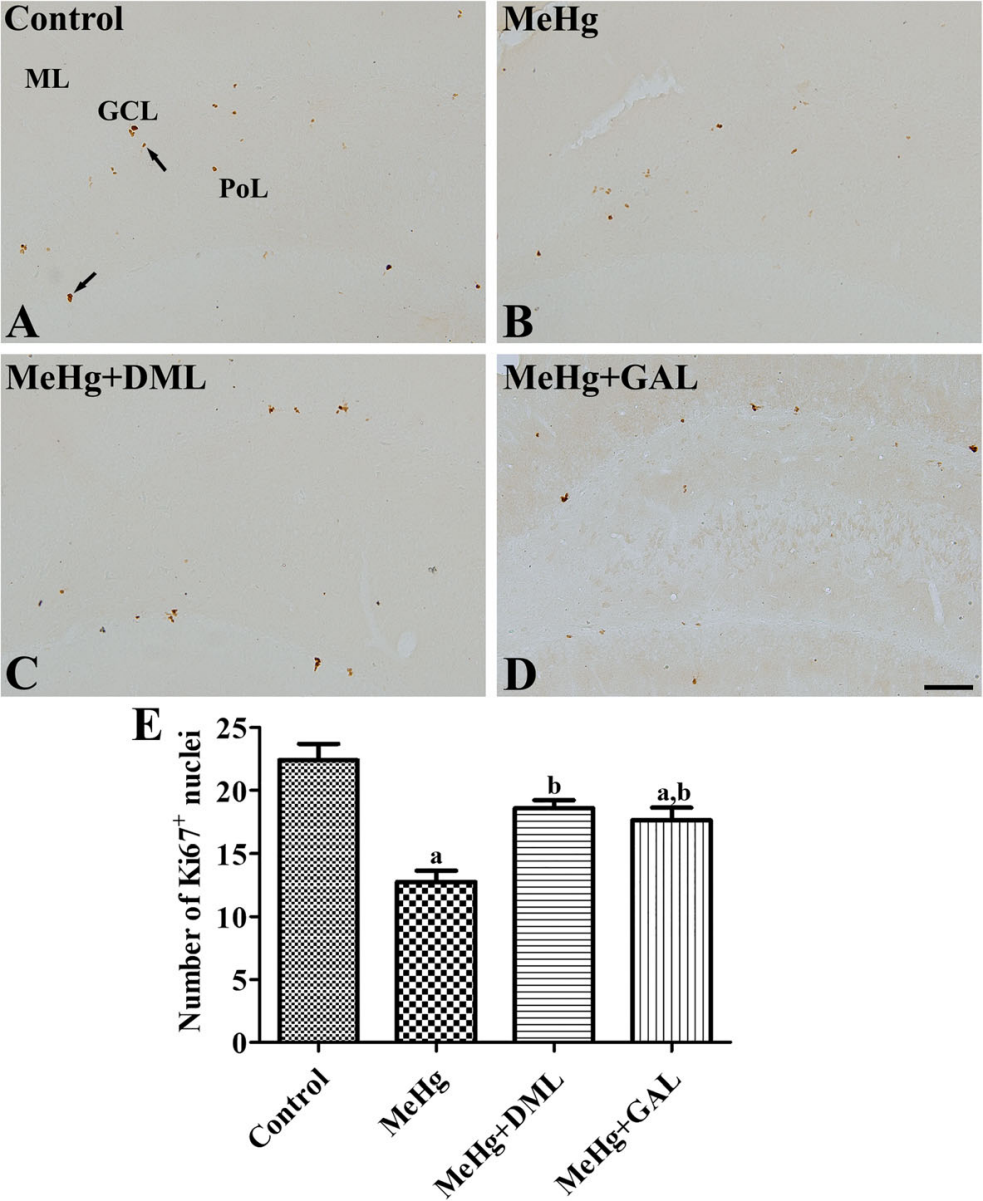
Immunohistochemistry for Ki67 in the dentate gyrus of the control group (**a**), dimethylmercury (MeHg)-treated group (**b**), Dendropanax morbifera leaf extract treated group with MeHg (MeHg + DML) (**c**), and galantamine (GAL) treated group with MeHg (MeHg + GAL) (**d**). In the control group, Ki67 positive nuclei (arrows) are mainly observed in the subgranular zone of the dentate gyrus. Note that few Ki67 positive nuclei are found in the MeHg-treated group, while in the MeHg + DML-treated group, Ki67 positive nuclei are relatively abundant. GCL, granule cell layer; ML, molecular layer; PoL, polymorphic layer. Scale bar = 100 μm. (**e**): Quantitative analysis of Ki67 positive nuclei number per section in the control, MeHg-treated, and MeHg+DML-treated rats using an image analyzer (n = 5 per group; ap <0.05, vs. control group; bp < MeHg-treated group). Values are mean ± standard error of the mean

In all groups, Ki67-positive nuclei were mainly observed in the subgranular zone of dentate gyrus (Fig. 3a-d). However, there were significant differences in the number of Ki67-positive nuclei in the dentate gyrus among groups (Fig. 3e). In the MeHg-treated group, the number of Ki67-positive nuclei was significantly decreased to 57.0% of that observed in the control group. However, in the MeHg+DML- and MeHg+GAL-treated groups, the numbers of Ki67-positive nuclei were significantly increased to 145.8 and 138.2% of that observed in the MeHg-treated group, respectively, although the number was significantly lower in the MeHg+GAL-treated group than in control group (Fig. 3).

### Effects of MeHg and DML on neuroblast differentiation in the dentate gyrus

**Fig. 4:**
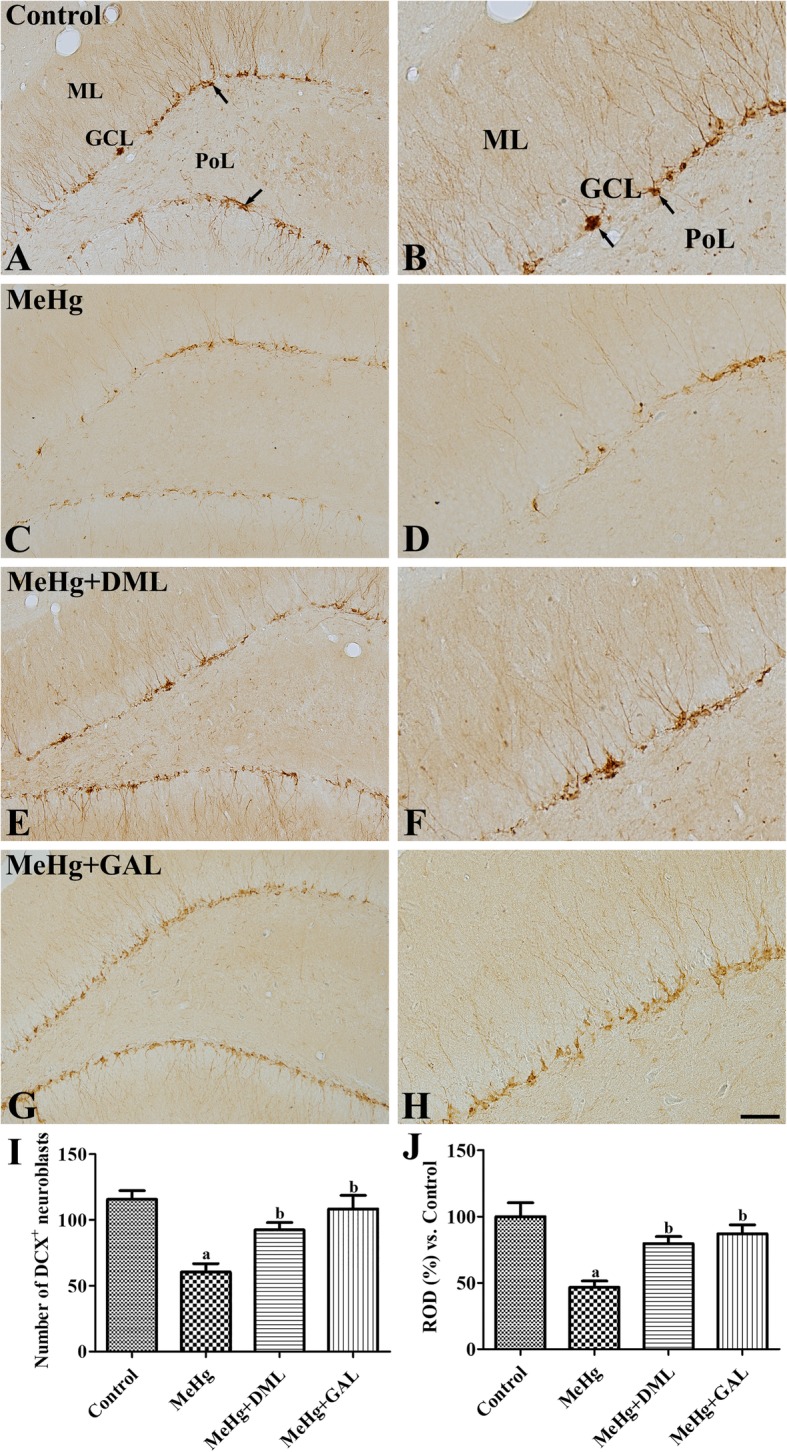
Immunohistochemistry for doublecortin (DCX) in the dentate gyrus of the control group (**a** and **b**), dimethylmercury (MeHg)-treated group (**c** and **d**), Dendropanax morbifera leaf extract treated group with MeHg (MeHg+DML) (**e** and **f**), and galantamine (GAL) treated group with MeHg (MeHg+GAL) (**g** and **h**). In the control group, DCX immunoreactive neuroblasts are abundantly in the dentate gyrus. Note that DCX immunoreactive neuroblasts are few and their dendrites are poorly developed in the MeHg-treated group. In the MeHg+DML-treated group, DCX immunoreactive neuroblasts are abundant and have well-developed dendrites. GCL, granule cell layer; ML, molecular layer; PoL, polymorphic layer. Scale bar = 100 μm (**a**, **c**, **e**, and **g**), 50 μm (**b**, **d**, **f**, and **h**). (**i** and **j**): Quantitative analysis of DCX immunoreactive neuroblasts. The number per section and the relative optical densities (RODs), expressed as a percentage of the value representing the DCX immunoreactivity in the dentate gyrus of the control group, are also shown (n = 5 per group; ap < 0.05, vs. control group; bp < MeHgtreated group). Values are mean ± standard error of the mean

In the control group, DCX immunoreactive cell bodies were found in the subgranular zone of the dentate gyrus, and their dendrites crossed the granule cell layer and extended into the molecular layer (Fig. 4a and b). The mean number of DCX immunoreactive neuroblasts in controls was 115.6 ± 14.9 per section (Fig. 4i). In the MeHg-treated group, fewer DCX immunoreactive neuroblasts and less DCX immunoreactive fibers were detected in the dentate gyrus than in the control group (Fig. 4c and d). In the MeHg-treated group, the number of DCX immunoreactive neuroblasts and DCX immunoreactivity were significantly decreased in the dentate gyrus by 52.3 and 46.7% than that of the control group, respectively (Fig. 4i and j). In the MeHg+DML- and MeHg+GAL-treated groups, more DCX immunoreactive neuroblasts were observed, with well-developed DCX immunoreactive dendrites than the MeHg-treated group (Fig. 4e, f, g, and h). In the MeHg+DML-treated group, the number of DCX immunoreactive neuroblasts and DCX immunoreactivity were significantly increased by 152.8 and 170.5% than that in the MeHg-treated group, respectively (Fig. 4i and j). In the MeHg+GAL-treated group, similar number and immunoreactivity of DCX positive neuroblasts were observed in the dentate gyrus compared to that in the MeHg+DML-treated group (Fig. 4i and j).

## Effects of MeHg and DML on AChE activity in hippocampal homogenates

**Fig. 5:**
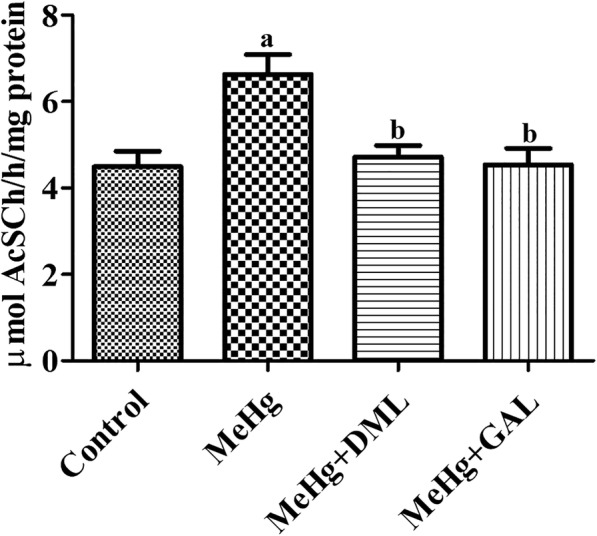
Acetylcholinesterase (AChE) activity in hippocampal synaptosomes of the control group, dimethylmercury (MeHg)-treated group, Dendropanax morbifera leaf extract treated group with MeHg (MeHg+DML) and and galantamine (GAL) treated group with MeHg (MeHg+GAL). Hippocampal AChE activity is expressed in μmol acetylthiocholine iodide (AcSCh)/h/mg of protein (n = 5 per group; ap < 0.05, vs. control group; bp < MeHg-treated group). Values are mean ± standard error of the mean. Note that AChE activity in the MeHg group is significantly increased compared to that in the control group, while this effect is significantly ameliorated in the MeHg+DML and MeHg+GAL group

In the control group, AChE activity was 4.50 μmol AcSCh/h/mg protein in hippocampal homogenates. In the MeHg-treated group, AChE activity was significantly increased by 147.4% of control group, while in the MeHg+DML- and MeHg+GAL-treated groups, AChE activity was significantly decreased by 71.2 and 68.5% of the MeHg-treated group, respectively, and was similar to the levels observed in the control group (Fig. 5).

## Discussion

MeHg shows toxic effects on human health, and prenatal exposure to mercury reduces the number of neuron and affects cytoarchitecture [31, 32]. Previous studies have shown that exposure to low or high levels of MeHg through the diet may accumulate in the hippocampus [4, 20, 33] because MeHg can easily cross the blood-brain barrier and then readily converted to an inorganic form in the brain [2, 3]. Studies have also demonstrated that early postnatal exposure to MeHg induces deficits in spatial memory [6, 7, 34]. In the present study, we observed that the administration of MeHg to adolescent to young-adult rats showed deficits in spatial memory in the Morris water maze test and novel object recognition test. In addition, supplementation with DML ameliorated the MeHg-induced impairments in the rats. We and our colleagues have previously demonstrated that DML or the root extract from *Dendropanax morbifera* ameliorated cadmium- [20] or D-galactose-induced [17] memory deficits in experimental animals, while an ethyl acetate fraction from *Dendropanax morbifera* mitigated high fat diet-induced cognitive impairments [35].

In the present study, we examined proliferating cells and differentiated neuroblasts in the dentate gyrus because stimulation and impairments of hippocampal neurogenesis are closely related to hippocampus-dependent learning and memory [36–38]. During the postnatal development of the hippocampus, exposure to MeHg (0.6 μg/g, subcutaneously) reduced hippocampal neurogenesis [39]. Similarly, MeHg exposure at P7 led to a marked decrease in the number of bromodeoxyuridine-positive cells by 29% in the granule cell layer and by 22% in the hilar region at P21 [6]. In the present study, treatment of rats with 5 μg/g MeHg significantly decreased proliferating cells and differentiated neuroblasts in the dentate gyrus, assessed by Ki67 and DCX immunohistochemistry, in adolescent/young-adult rats. In addition, the supplementation with DML significantly ameliorated the MeHg-induced reduction of proliferating cells and differentiated neuroblasts in the dentate gyrus. In a previous study, we demonstrated significant increases in these cells in the dentate gyrus after treatment with the root extract from *Dendropanax morbifera* in normal (naïve) mice; furthermore, supplementation with *Dendropanax morbifera* ameliorated the cadmium-induced reduction in proliferating cells and differentiated neuroblasts [20].

AChE catalyzes the breakdown of acetylcholine and inhibition of AChE is a major target for Alzheimer’s disease. In the present study, we also measured AChE activity in hippocampal synaptosomes. Exposure to MeHg produced significantly higher levels of AChE activity compared to that in the control group. This result is consistent with a previous study showing that subchronic treatment with mercury chloride increased AChE activity in the hippocampus by 125% than that in the control group [40]. Supplementation with DML significantly decreased MeHg-induced activation of AChE activity in hippocampal synaptosomes. This result is consistent with that of a previous study demonstrating that administration of an ethyl acetate fraction from *Dendropanax morbifera* lowered AChE activity compared with that in the high-fat diet fed group [35]. Furthermore, inhibition of AChE by donepezil or GAL increased proliferating cells or hippocampal neurogenesis in the dentate gyrus of vascular dementia rats or normal mice [41–43]. In the present study, we confirmed that GAL also reduced MeHg-induced memory impairments and reduction of proliferating cells and differentiated neuroblasts in the dentate gyrus and DML has comparable effects on hippocampal functions compared to that in the GAL.

In the present study, we also analyzed the contents of phenolic compounds by HPLC system demonstrated by Hyun et al. [24]. They observed analyzed 22 phenolic compounds in DML from green and senescent leaves [24] and we selected most abundant polyphenols in the present study and observed rutin and chlorogenic acid are most abundant in the DML. This result was supported by previous studies that rutin could decrease MeHg bioaccessibility in vitro digestion experiment [44]. Chlorogenic acid protects hippocampal neurons from aluminum-induced cytotoxicity by antioxidant actions [45]. In addition, chlorogenic acid ameliorates the scopolamine-induced amnesia in mice by anti-AChE and antioxidant activities [46]. In the present study, we used 80% ethanol solution for extraction of DML and Choi et al. observed highest levels of rutin and chlorogenic acid at 80% ethanol extracts [47]. In addition, they observed highest scavenging and reducing power activities at 80% ethanol extracts of DML [47]. In other components, myricetin, although the lowest contents in six polyphenols in DML, has antioxidant effects of MeHg-induced oxidative stress such as formation of reactive oxygen species and lipid peroxidation [48].

## Conclusions

DML ameliorated MeHg-induced memory deficits, an effect that was associated with DML-induced cell proliferation and neuroblast differentiation, as well as lowered AChE activity, in the hippocampus.

## Abbreviations

AChE: Acetylcholinesterase
AcSCh: Acetylthiocholine iodide
DCX: Doublecortin
DML: Extract of *Dendropanax morbifera* leaves
GAL: Galantamine
HPCL: High-performance liquid chromatography
IP: Intraperitoneal
MeHg: Dimethylmercury
P7: Postnatal day 7
PBS: Phosphate-buffered saline
ROD: Relative optical density
